# Quantitative and Phylogenetic Analyses of Immature Neurons in Cortical Layer II and Amygdala of Macaque Monkeys

**DOI:** 10.3390/cells15131158

**Published:** 2026-06-25

**Authors:** Alessia Pattaro, Marco Ghibaudi, Madeline Bramel, Chet C. Sherwood, Luca Bonfanti

**Affiliations:** 1 Neuroscience Institute Cavalieri Ottolenghi, 10043 Orbassano, Italy; alessia.pattaro@unito.it (A.P.); mag908@pitt.edu (M.G.); 2 Department of Veterinary Sciences, University of Turin, 10095 Grugliasco, Italy; 3 Department of Anthropology and Center for the Advanced Study of Human Paleobiology, The George Washington University, Washington, DC 20052, USA; mbramel@gwmail.gwu.edu

**Keywords:** brain plasticity, cerebral cortex, doublecortin, arrested maturation, evolutionary trade-off, primates

## Abstract

**Highlights:**

**What are the main findings?**

- Cortical and subcortical immature neurons were quantified in rhesus macaques to be compared with other mammals previously analyzed with the same method and to put results in a phylogenetic context.
- Immature neuron densities of macaques were found to be aligned with other gyrencephalic species in the cerebral cortex and with primates in the amygdala.

**What are the implications of the main findings?**

- Immature neurons are a prevalent form of structural plasticity that has arisen independently in evolution among diverse large-brained, gyrencephalic mammals.
- Macaque monkeys can be a model of immature neurons for the translation of results in humans.

**Abstract:**

“Immature” or “late-maturing” neurons exist in layer II of the cerebral cortex (cortical immature neurons; cINs) and within the amygdaloid complex (subcortical immature neurons; scINs). These cells remain in a prolonged state of arrested development yet retain the ability to resume maturation and to functionally integrate into neural circuits. Both cINs and scINs are abundant in large-brained mammals with respect to small-brained, lissencephalic rodents. In previous reports, using a comparable method for quantification in diverse mammals, including mice, chimpanzees, and other species, we showed positive correlation of immature neuron cell density with brain size and gyrencephaly. Here, we quantified the cINs and scINs in the cerebral cortex and amygdala of young adult rhesus macaques to determine how they compare to phylogenetic variation. Our results further demonstrate the existence of covariance between cIN density and both increasing brain size and neocortical expansion, as well as the specialized increase of scINs in the amygdala of primates. These findings support the emerging view that immature neurons may represent a reservoir of undifferentiated (stem cell-independent) neuronal cells for the widely expanded cortices and amygdala of mammals endowed with high-order cognitive functions and complex sociality. The detailed mapping of cortical and subcortical immature neurons in a primate often used in translational research sets the foundation for deeper, functional studies aimed at understanding human brain plasticity.

## 1. Introduction

**Figure 1:**
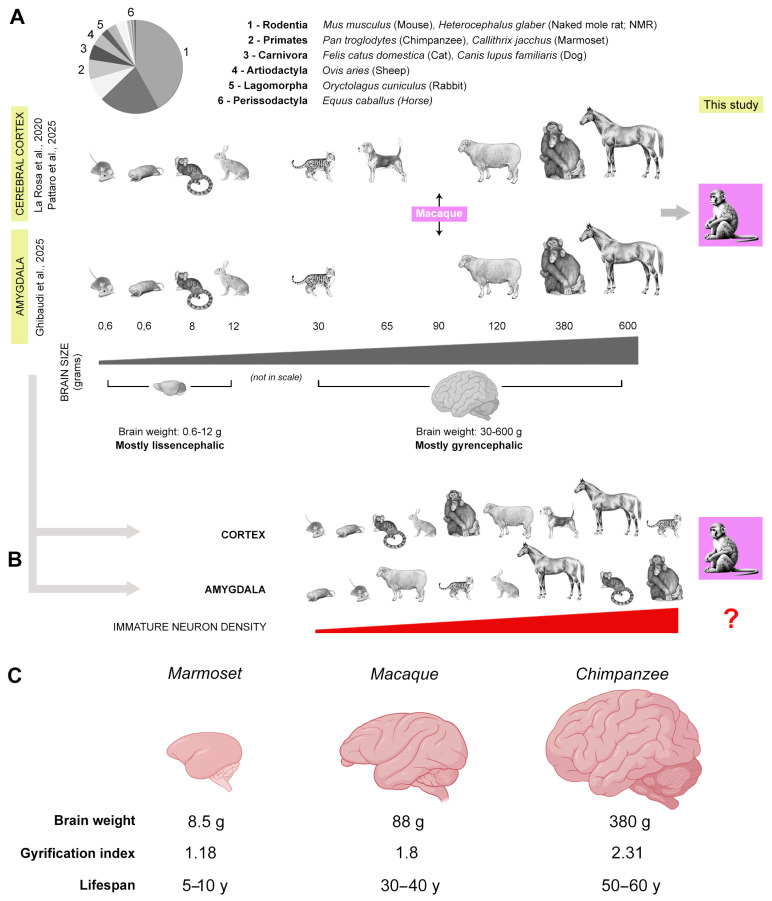
Phylogenetic variation of cortical and subcortical immature neurons, as previously reported. (**A**) Top, pie chart: distribution of mammal species across orders, based on [27]; numbers indicate the position of the different orders. Bottom, animal species previously used to study the interspecies variation of cortical INs (cINs [15,16]) and amygdala INs (subcortical immature neurons, scINs [17]). Icons of the species included correspond to those quantified in young adult stages in the two regions, and thus are fully comparable, apart from marmosets used for cortex analysis, which were referred to as adults. Both cINs and scINs are quite rare in small-brained, lissencephalic species (e.g., rodents), while very abundant in gyrencephalic mammals within the neocortex and in primates within the amygdala. Animal species are arranged from left to right according to increasing brain size (in (**A**)) and to increasing IN density (in (**B**)). (**C**) Among primates, the brains of macaques occupy an intermediate position between common marmosets and chimpanzees regarding brain size, gyrification, and lifespan, thus representing a valuable translational model for humans. Animal icons in (**A**,**B**) were reproduced with permission from [15]; this article is distributed under the terms of the Creative Commons Attribution License, which permits unrestricted use and redistribution provided that the original author and source are credited, except for the macaque icon, which was generated with AI. Icons in (**C**) were created with BioRender.

Recently, a neuronal population referred to as “immature”, “late-maturing”, or “dormant” neurons has become increasingly recognized as an important contributor to brain structural plasticity (hereafter referred to as immature neurons, INs) [1,2,3,4,5,6,7,8,9]. INs are prenatally generated cells whose maturation is arrested, thus expressing typical markers of immaturity (e.g., the cytoskeletal protein doublecortin—DCX [10], and the polysialylated form of the neural cell adhesion molecule—PSA-NCAM [9]) during variable periods of the lifespan, depending on the animal species. They were first described in the piriform cortex of rats (cortical immature neurons, cINs; history summarized in [9]) and very similar cells were found in the amygdala (subcortical immature neurons, scINs [11,12,13,14]). In previous reports, we examined brains of diverse mammals (Figure 1A) to show that both cINs and scINs display marked interspecies variation, resulting in far more abundance in large-brained, gyrencephalic species in comparison to rodents [13,15,16,17] (Figure 1B). Accordingly, we proposed that a trade-off between different types of neurogenic plasticity has occurred during evolution (stem cell-driven neurogenesis versus maintenance of immature neurons), leading rodents to mostly rely on hippocampal and olfactory bulb adult neurogenesis while large-brained species invest more in late-maturing neurons in the cortex and amygdala [18,19,20]. The occurrence of undifferentiated neurons in high-order cognitive regions devoid of stem cell-driven neurogenesis might be of utmost importance for future translational outcomes in humans, considering brain development and sculpting, aging, and disease [20,21,22,23].

Although the existence of INs has been confirmed in humans, in both the cerebral cortex [24,25] and amygdala [14], no systematic quantitative analyses are available. Using an approach previously established for obtaining comparable quantitative results in phylogenetically diverse brains [26], new mammal species can be added in the context of the variation that has already been established (see for example [16]). Among the species analyzed and fully quantified previously (10 for the cortex, 8 for the amygdala, including small, lissencephalic and large, gyrencephalic mammals), two different primates have been studied (Figure 1B): common marmosets (*Callithrix jacchus*) and chimpanzees (*Pan troglodytes*). Here, the cINs and scINs were quantified in the brains of young adult rhesus macaque monkeys (*Macaca mulatta*) by using the same approach, allowing us to place them in the range of interspecies variation and to establish a comparison with other primates. Concerning brain size, gyrification, and lifespan, macaques occupy an intermediate position between marmosets and chimpanzees (the two primate species for which we know the immature neuron density in both cortex and amygdala [15,17]; Figure 1C).

Although most biomedical research utilizes rodent models, nonhuman primates are important because of their similarity to humans in physiology, neuroanatomy, reproduction, development, cognition, and social complexity [28,29,30,31]. Here, we focused on the number and distribution of cINs and scINs in the brains of rhesus macaques to extend our interspecies analysis aimed at defining the occurrence and phylogenetic variation of this cell population in mammals. These results provide foundational information for future studies investigating the cellular characteristics and functions of primate INs.

## 2. Materials and Methods

### 2.1. Brain Sample

Rhesus macaque monkey brains used in this study were collected from three sources: JBSA Fort Sam Houston, Emory National Primate Research Center, and Oregon National Primate Center. All necessary authorizations were provided, and all experiments were conducted in accordance with current US laws (see Ethics Statement).

Young adult macaques (4 males; age: 7–10 years; Table 1) were euthanized for reasons unrelated to the current study and obtained through tissue distribution programs. Brains were extracted with a post-mortem interval (PMI) of less than 1 h and fixed by immersion in 10% buffered formalin and kept in fixative solution for at least one week. Then, brains were stored in PBS containing 0.1% sodium azide at 4 °C until use.

### 2.2. Tissue Processing for Histology and Immunohistochemistry

**Figure 2:**
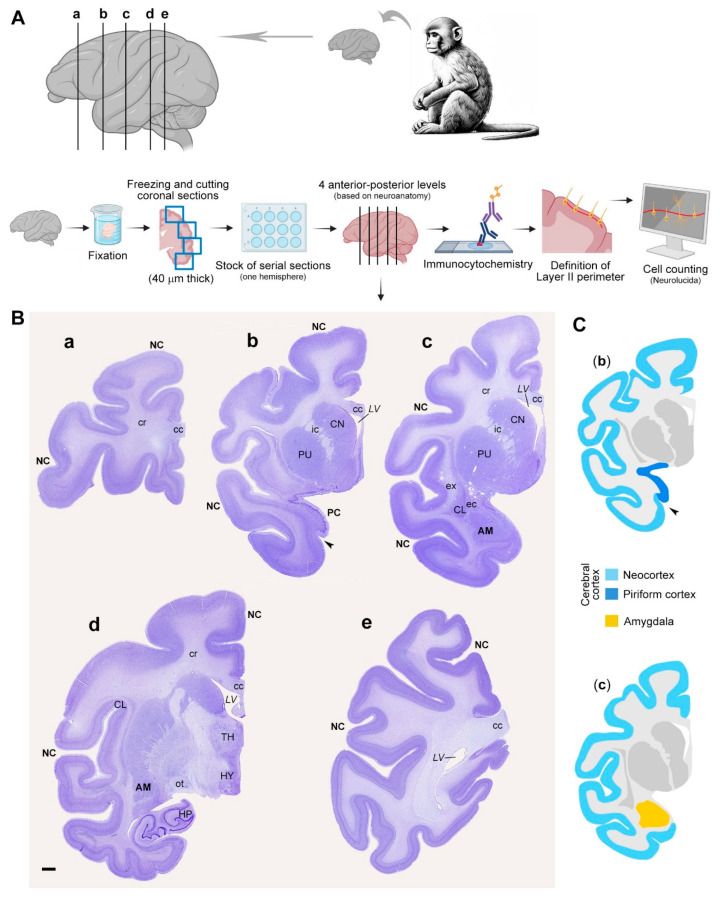
Neuroanatomical references of the macaque brains used in this study. (**A**) Subsequent steps from brain extraction to cell quantification, including fixation; cutting serial coronal sections of the same thickness in the whole hemisphere; immunocytochemical procedures; layer II length (or amygdala area) measuring; and cell counting at the Neurolucida. (**B**) Mini-atlas of the main neuroanatomical structures, based on coronal Nissl-stained sections (**a**–**e**). The macaque atlases by [32,33,34,35,36,37] were used for comparison. Abbreviations: capital letters, gray matter main structures; lowercase letters, white matter tracts; italics, main cavities; bold, regions considered in this study for the analysis of immature neurons (NC, neocortex; PC, piriform cortex; LV, lateral ventricle; cc, corpus callosum; cr, corona radiata; ic, internal capsule; ec, external capsule; ex, extreme capsule; ot, optic tract; HY, hypothalamus; TH, thalamus; CN, caudate nucleus; PU, putamen; AM, amygdala; CL, claustrum; HP, hippocampus). The transition between three-layered allocortex (piriform cortex) and six-layered isocortex (neocortex) is marked with arrowheads. Macaque icon generated with A.I. (**C**) Schematic representation of the cortical (**b**) and subcortical (**c**) regions investigated for the DCX^+^ immature neurons. Scale bar: 2000 µm. Brain icons created with BioRender.

The left hemisphere for each animal was dissected coronally in slabs (1–1.5 cm thick) and placed in sucrose solutions of gradually increasing concentration up to 30% in 0.1 M phosphate-buffered saline solution (PBS) pH 7.4 at 4 °C for dehydration and cryoprotection. Then, the tissues were frozen by immersion in dry ice, sectioned with a microtome in coronal sections (40 µm thick) and stored as free-floating sections in an anti-freezing solution at −20 °C until use (Figure 2A).

The sections were used both for histological staining procedures (Nissl staining, aimed at defining the overall neuroanatomy to identify the corresponding brain structures; Figure 2) and for immunocytochemical detection of the immaturity marker doublecortin (DCX).

For 3,3-diaminobenzidine (DAB) immunohistochemistry (peroxidase technique), free-floating sections were rinsed in 0.01 M PBS, pH 7.4. Antigen retrieval was performed using citric acid, pH 6.0, at 90 °C for 30 min. After further washing in 0.01 M PBS, the sections were immersed in an appropriate blocking solution (3% Bovine Serum Albumin (MP Biomedicals, Irvine, NA, USA), 5% Normal Goat Serum (Vector laboratories, Newark, CA, USA), 1% Triton X-100 (Sigma-aldrich, Saint Louis, MO, USA) in 0.01 M PBS) for 90 min at room temperature (RT). Then, the sections were incubated with the primary antibody (polyclonal rabbit anti-DCX antiserum, Cell Signaling, code 4604–RRID AB_561007; raised against the antigenic sequence that surrounds amino acid 350 tyrosine of human DCX; dilution: 1:600) for 48 h at 4 °C. After washing in 0.01 M PBS, the sections were incubated for 2 h at RT with biotinylated secondary antibody (anti-rabbit made in goat, 1:250, Vector Laboratories), followed by three washes in 0.01 M PBS, and incubated in an avidin–biotin–peroxidase complex (Vectastain ABC kit; Vector Laboratories, Burlingame, CA, USA, PK-4000) for 1 h at RT. The reaction was detected with DAB as a chromogen in 50 mM TRIS-HCl, containing 0.025% hydrogen peroxide, for few minutes and then washed in 0.01 M PBS. The sections were counterstained with Nissl staining, mounted with Permount Mounting Medium (Fisher Chemical, Waltham, MA, USA, SP15-500) and coverslipped.

### 2.3. Brain Neuroanatomy

Working atlases for each brain in the study were constructed using Nissl-stained serial coronal sections (Figure 2B) to identify the main neuroanatomical regions. These specimens were compared with existing macaque brain atlases [32,33,34,35,36,37], which constituted valuable references.

Both the cerebral cortex (including its main subdivisions of piriform cortex and neocortex, here intended as the rest of the cortex or cortical mantle, excluding the hippocampus; Figure 2C) and amygdala (Figure 2C) were investigated by using the same protocol employed in our previous studies (a summary is given below; for more details, see [15,16,17,26]).

**Figure 3:**
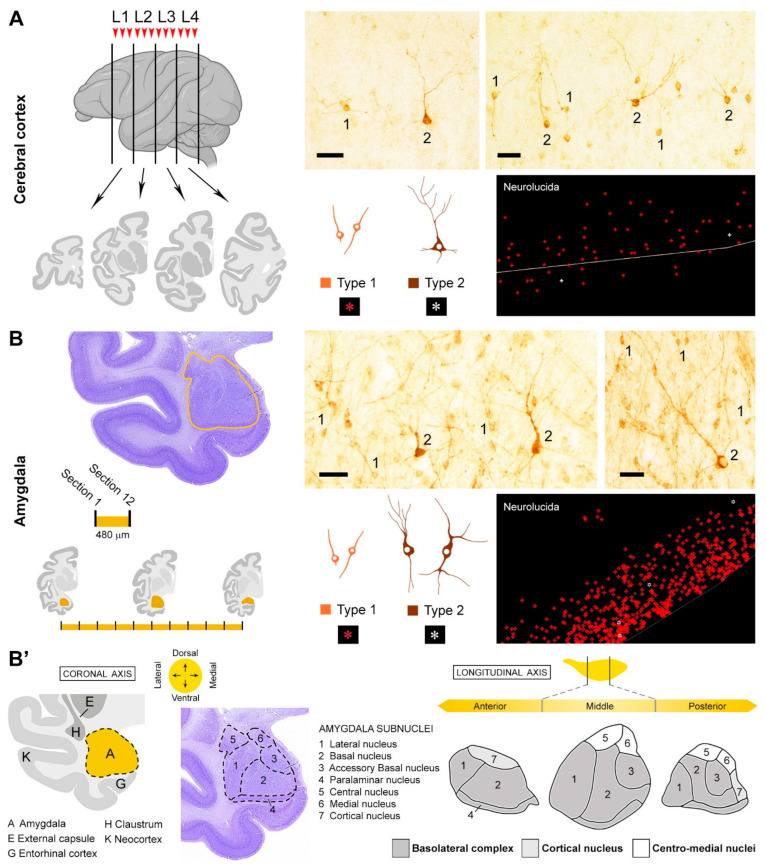
Methodological approach for processing different brains to obtain comparable results in the cerebral cortex and amygdala (see [26] for more details). (**A**) Method adapted for IN quantification in cerebral cortex layer II [15]; twelve coronal sections obtained from 4 corresponding brain levels are considered. (**B**) Method adapted for IN quantification in the amygdala [17]; serial coronal sections spaced 480 µm (one out of twelve) are considered along the total length of the amygdala (11 sections in a macaque hemisphere). In both regions, type 1 and 2 cells, corresponding to different maturational stages, can be identified and counted. Scale bars: 30 µm. (**B’**) Topological location of the macaque amygdala and its subdivision into subnuclei. Brain icon in (**A**) created with BioRender.

For the analysis of IN in the cortex, four anterior-to-posterior coronal brain levels (referred to as L1–L4; Figure 3A) were established based on corresponding neuroanatomical structures: L1, starting from the beginning of the lateral ventricle (+33 mm rostral to the ear bar zero–EBZ or interaural in macaques); L2, starting from the internal capsule; L3, starting from the beginning of the amygdala; and L4, extending from the closure of the lateral ventricle to a thickness equivalent to that of L2, corresponding to the same number of serial sections (section thickness = 40 µm) [15,16,26]. These neuroanatomical references allowed comparisons among cerebral cortices of brains across a range of sizes and gyrencephaly. For technical reasons, partly related to interindividual differences, a few sections at the most anterior part of the frontal pole and at the most posterior part of the occipital pole were excluded during sectioning.

For the analysis of INs in the amygdala, serial sections of the entire subcortical region (spaced apart from each other by 480 µm) were used (Figure 3B). After having established the topology and orientation of the amygdaloid complex along its full anterior-posterior axis, including its anatomical relationships with surrounding structures (Figure 3B’), the amygdala was also divided into its subnuclei, identified by comparing Nissl-stained serial coronal sections with existing neuroanatomical atlases and comparative studies from the literature [32,33,34,35,36,37]. The same serial section number was considered for all animals, using the specimen with the lowest number of sections as a reference to standardize the total number of sections across specimens [15]. The subnuclei were grouped into three major divisions: the basolateral complex (comprising the lateral, basal, and accessory basal nuclei), the centro-medial complex (including the central and medial nuclei), and the cortical nucleus.

On average, a total of 1750 coronal sections were cut in each hemisphere (see Dataset S1). Twelve sections (three per each brain level; see above) were used for cell counting in the cortex, and eleven were used for cell counting in the amygdala (see above). A total of 92 coronal sections (48 in the cortex; 44 in the amygdala) were used for quantitative analysis of INs. Other sections were used for histological staining aimed at defining neuroanatomy.

### 2.4. Image Acquisition and Processing

Images were collected using an Axioplan 2 imaging microscope (Carl Zeiss Microscopy, White Plains, NY, USA). For Nissl-stained sections of the mini-atlases, an Epson Perfection V600 Photo scanner was used. All images were processed using ImageJ (version 1.54p, Rasband, W.S., ImageJ, U. S. National Institutes of Health, Bethesda, MD, USA) and Photoshop CS4 (Adobe Systems, San Jose, CA, USA). Only general adjustments to color, contrast, and brightness were made.

### 2.5. Quantitative Analysis

Analyses of cINs were conducted within cortical layer II considering four anterior–posterior brain levels (L1-L4; Figure 3A), established using corresponding neuroanatomical structures to obtain comparable results, as previously reported [15,16,26] (Figure 2). DCX^+^ cell counting was performed on the entire cerebral cortex layer II perimeter (3 coronal sections/level, including both the piriform cortex and the neocortex, while excluding the archicortex) by marking type 1 and 2 cells separately (Figure 3A). Analyses of scINs were performed on the entire extent of the amygdala, as previously described [17] (Figure 3B). A fixed number of serial coronal sections of the amygdala was considered. In particular, the total number of sections was compared across specimens, and the sample with the lowest section number was used as a reference. Accordingly, sections at the beginning and at the end of the amygdala were excluded to match this number across all specimens, as was performed previously [17].

Quantification was conducted on sections stained for DCX with the DAB technique and quantified using direct cell counting with Neurolucida software (version 2024.1.3, MBF Bioscience, Colchester, VT), to be consistent with our previous analysis aimed at obtaining fully comparable results [26]. The perimeters of the cortex (piriform cortex + neocortex) and amygdala were traced at low magnification (4×) using the “Contour” tool of Neurolucida. All DCX^+^ cells were counted (direct cell counting) with markers using a 20× magnification objective lens [26]. In the cortex, results were expressed as linear density (number of DCX^+^ cells/mm of layer II perimeter), while the entire amygdala area was considered in subcortical analyses (number of DCX^+^ cells/mm^2^ amygdala area). Cells cut on the superior surface were excluded to avoid overcounting. Cell soma sizes were obtained by evaluating the cell soma width (diameter orthogonal to the main axis), measured in about 100 cells/region in each sample using the Neurolucida ‘measure line’ tool.

### 2.6. Statistical and Phylogenetic Analysis

All graphs and statistical analyses were performed using GraphPad Prism Software (version 8.0.2, San Diego, CA, USA). Data distribution within each group was assessed using the Shapiro–Wilk normality test. Since our data were not normally distributed, we used nonparametric tests: the Kruskal–Wallis test with Dunn’s multiple comparison post hoc test, and the Mann–Whitney test. Medians were used as the central measure of data, as previously described [15,16,17,26]. A mixed-effects analysis, followed by Tukey’s multiple comparisons test, was also performed on the linear density values obtained from each section. A *p* < 0.05 was considered statistically significant.

Species median DCX^+^ cell densities were used to perform ancestral character state reconstructions of trait evolution mapped onto the phylogeny. First, a phylogenetic tree of the investigated species (macaques analyzed in this project and data from other species reported in [15,16,17]) was downloaded from the TimeTree database (access date: 26 February 2025) [38]. Then, ancestral character state reconstruction was implemented in Mesquite software (version 3.81, Mesquite Project, Vancouver, BC, Canada), using a parsimony model.

The association between DCX^+^ cell density and brain size and neocortical surface was investigated. All data were log-transformed to fit power functions for least squares regression, in accordance with standard procedures in comparative neuroanatomy studies. Although we also performed phylogenetic generalized least squares (PGLS) regression to control for potential phylogenetic non-independence, Pagel’s λ was estimated at zero, suggesting the absence of a detectable phylogenetic signal in the dataset. Therefore, ordinary least squares regression was used, and these analyses were performed on the Statistics Kingdom website (statisticskingdom.org, access date: 26 February 2025).

## 3. Results

### 3.1. Features of Immature Neurons and Comparison with Previous Reports

To obtain quantitative results that are comparable with previous analyses, the same experimental procedures were adopted and coronal sections for cell counting were chosen at the same corresponding neuroanatomical levels (method explained in detail in [26]). As in previous reports [15,16,17], in each hemisphere, 12 coronal sections from 4 anterior-posterior levels (3 sections/level) were used for quantification in the macaque cerebral cortex, while 11 coronal sections spaced 480 µm from each other were used for the amygdala (Figure 3). This allowed us to perform DCX^+^ cell counting in the same conditions and at homologous brain levels used for other smaller and larger mammal species.

After immunocytochemistry, typical DCX^+^ cells were found at their specific locations, namely the cerebral cortex layer II (including the piriform cortex and neocortex, as expected in a gyrencephalic species [15,16]) and in the basolateral amygdala [17]. The main features of INs, as a population of undifferentiated DCX^+^ cells conserved across widely different mammals, are now well defined at both cortical and subcortical locations [4,6,13,15,16,17,39,40]. They consistently occur in two main morphologies (Figure 3A,B): small, unipolar/bipolar cells (type 1 cells) and large, highly ramified cells (type 2 cells) corresponding to the principal neuronal type of cortical layer II (cINs, in both the piriform cortex and neocortex of gyrencephalic species) and of the basolateral amygdala (scINs). Previous studies carried out on at least 8 diverse mammals reported an average cell soma diameter ranging between 3 and 9 µm for type 1 and between 7 and 20 µm for type 2 cells in the cerebral cortex, and between 3 and 9 µm for type 1 and between 9 and 19 µm for type 2 cells in the amygdala (for measures referring to each animal species, see Table S1 [15,16,17]).

**Figure 4:**
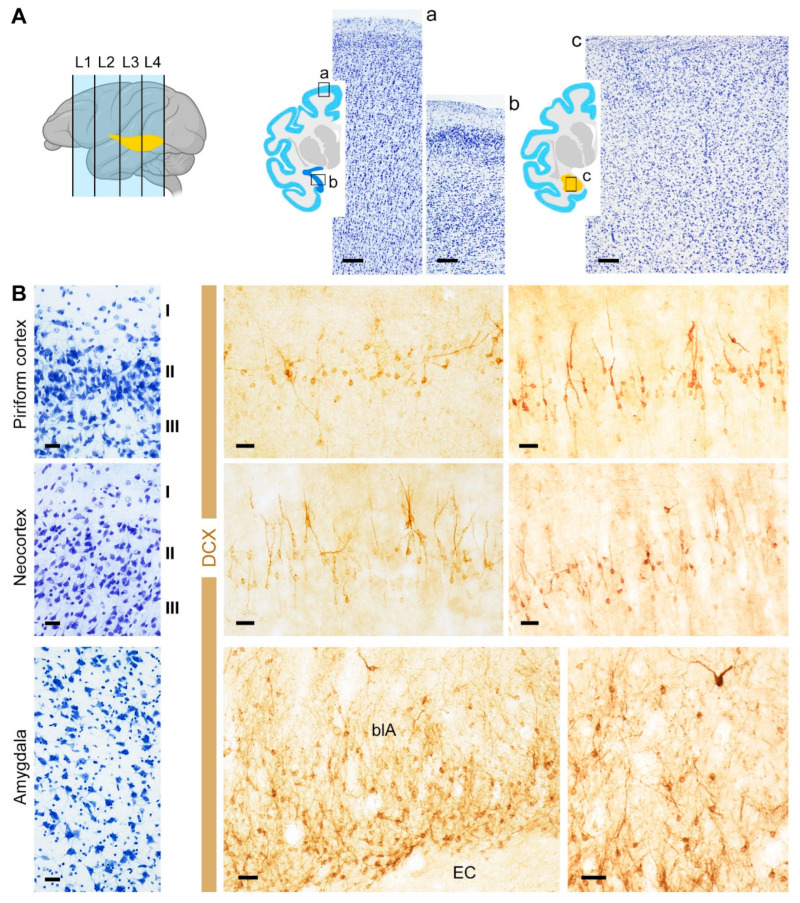
Once the regions of interest were defined through histological analysis (**A**; see Figure 2 and Figure 3; a, neocortex; b, piriform cortex; c, amygdala), the immunostaining for DCX in the cerebral cortex (piriform cortex and neocortex) and amygdala reveals typical populations of immature neurons (**B**). Photographs refer to layer II in the cortex and to the basolateral complex in the amygdala (blA). EC, external capsule. Scale bars: (**A**) 200 µm; (**B**) 30 µm. Brain icon created with BioRender.

Multiple reports dealing with non-rodent mammal species consistently found DCX^+^ cells displaying the above-mentioned characteristics [13,14,15,16,17,39] and the same features were detectable in the macaques analyzed here (Figure 4; see below). In the cortex they were present in layer II of the piriform cortex and along the entire neocortical perimeter (Figure 4B), while in the amygdala they were observed in the basolateral complex (Figure 4B). Thus, the immature cells were systematically quantified in both the cerebral cortex and amygdala of macaques.

### 3.2. Quantification and Phylogenetic Analyses of Immature Neurons in Cerebral Cortex Layer II

**Figure 5:**
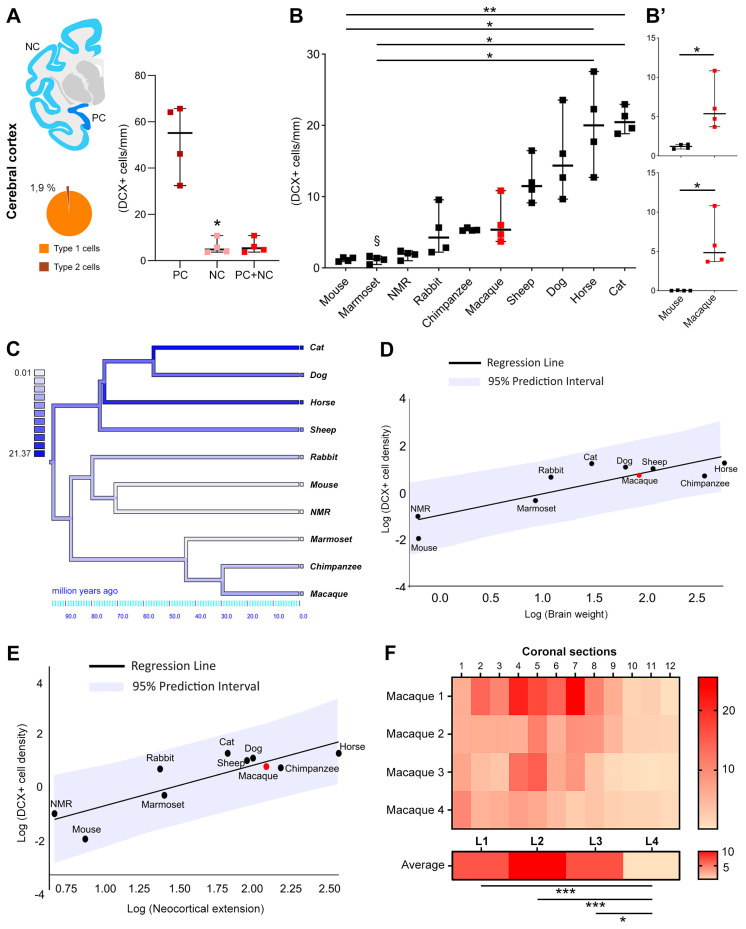
Cell densities and phylogenetic analysis of DCX^+^ immature neurons in the macaque cortex. (**A**) DCX^+^ cell densities in the piriform cortex (PC), neocortex (NC) and whole cerebral cortex (PC+NC, intended as piriform cortex+neocortex) of young adult macaques; top right, percentages of type 1 and type 2 cells in cortical layer II. (**B**) Positioning of macaque in the phylogenetic variation of cIN density in the cerebral cortex of mammals. Data obtained in the present study (in red) are compared with previously reported data (in black [15,16], all referring to young adults, except for adult marmosets, §). Macaques have among the highest densities, and a significant difference is found between rodents and gyrencephalic species (see direct comparison between mouse and macaque in (**B’**); top, whole cerebral cortex; bottom, neocortex). NMR, naked mole rat. (**C**) Ancestral character state reconstructions of trait evolution for DCX^+^ cell density in the neocortex mapped onto the phylogeny. Note that cat, dog, horse, and sheep, which exhibit the highest cIN density, belong to the clade Laurasiatheria (top), while rabbits, rodents and primates are together within the clade Euarchontoglires (bottom). (**D**) Least squares regression of DCX^+^ cell density in the neocortex against brain size (brain weight). (**E**) Least squares regression of DCX^+^ cell density in the neocortex against neocortical surface (calculated using the layer II perimeter). All regression plots are on a log scale and show the 95% prediction intervals. (**F**) Heatmaps of the distribution of DCX^+^ cell linear density in different brain levels (L1—L4; neocortex). * *p* < 0.05, ** *p* < 0.01, *** *p* < 0.001.

Counting of DCX^+^ cINs in the cerebral cortex of macaques was performed on a total of 48 brain coronal sections (on average 149 cm of layer II perimeter in each hemisphere, for a total of 596 cm in four animals) by considering the piriform cortex and neocortex separately (Dataset S1). The results are reported in Figure 5A. The cIN linear density in the whole cerebral cortex was close to that previously reported for chimpanzees (Figure 5B; see Section 4), and a similar result was obtained by considering only the neocortex (Figure S1). A higher cell density was observed in the piriform cortex (Figure 5A). A direct comparison between macaques and mice (data from [15]) was performed using a Mann–Whitney U test, revealing statistically significant differences in both the total cortex (*p* = 0.0286) and the neocortex (*p* = 0.0286) (Figure 5B’). The average cell soma diameters measured 3–8 µm for type 1 cells and 8–13 µm for type 2 cells. Differential counting of the two cell types revealed a vast majority of type 1 cells, with type 2 (complex) cells comprising around 2% (pie chart in Figure 5A). The DCX^+^ cells were detectable in the entire extent of the cerebral cortex considered in the four anterior-to-posterior brain levels, thus confirming previous findings of a consistent cIN presence throughout the neocortex of gyrencephalic species and other primates [15,16,24]. A heatmap analysis of the linear densities across anterior-to-posterior brain levels (L1-L4) revealed a widespread presence of cINs in all levels, with a higher density in level 2 and a lower density in level 4 (Figure 5F). The statistical analysis between the four brain levels confirmed a significant difference between L4 and all other levels (mixed-effects analysis with Tukey’s multiple comparisons; *p* < 0.05; lower cell density in L4: L1 vs. L4: *p* = 0.0002; L2 vs. L4: *p* = 0.0004; L3 vs. L4: *p* = 0.0427). The estimation of the total number of cINs in a whole macaque cerebral cortex (one hemisphere; obtained by multiplying the median density by the total number of coronal sections cut in each animal) reached around 1 million (1,060,500 for the cerebral cortex; 1,036,000 for the neocortex).

Linear densities obtained from systematic quantification of the DCX^+^ cells were used to perform phylogenetic and linear regression analyses (Figure 5C–E). Ancestral character state reconstructions of trait evolution for DCX^+^ cell density mapped onto the phylogeny was performed in the whole cerebral cortex (Figure S2) and neocortex (Figure 5C). Among the examined gyrencephalic brains, the clade Laurasiatheria (Carnivora, Artiodactyla, and Perissodactyla) exhibits the highest cIN density. Within the clade Euarchontoglires (Rodents and Primates), rodents show the lowest cIN density, with their divergence from primates occurring approximately 90 million years ago.

To explore the scaling relationship between DCX^+^ cell densities with brain size (cIN densities against brain weight; Figure 5D), least squares regression analyses were conducted using the data obtained here for macaques, compared with datasets previously reported for other young-adult mammals [15,16]. The results indicated a highly significant positive association for the neocortex (R^2^ = 0.75, *p* = 0.001; Figure 5D), and a significant positive association for the whole cerebral cortex (R^2^ = 0.57, *p* = 0.011; Figure S3).

The same regression analysis was performed for DCX^+^ cell densities with neocortical surface (cIN densities against neocortical extension, calculated by using layer II perimeters), also in this case confirming the existence of significant positive covariance (R^2^ = 0.73, *p* = 0.002; Figure 5E).

### 3.3. Quantification and Phylogenetic Analyses of Immature Neurons in the Amygdala

**Figure 6:**
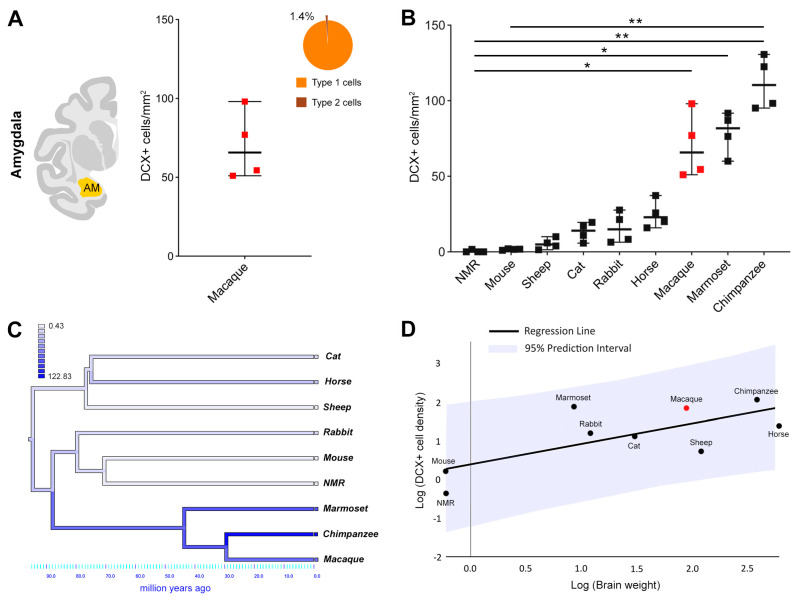
Cell densities and phylogenetic analysis of DCX^+^ immature neurons in the macaque amygdala. (**A**) DCX^+^ cell densities in the amygdala (AM) of young adult macaques; top right, percentages of type 1 and type 2 cells. (**B**) Positioning of macaque in the phylogenetic variation of cIN density in the amygdala of mammals. Data obtained in the present study (in red) are compared with previously reported data [17] (in black). Macaques have among the highest densities, along with other primates, while a significant difference is maintained with other species, especially rodents. NMR, naked mole rat. (**C**) Ancestral character state reconstructions of trait evolution for DCX^+^ cell density mapped onto the phylogeny in the amygdala. (**D**) Least squares regression of DCX^+^ cell density in the amygdala against brain size (brain weight). The regression plot is on a log scale and shows the 95% prediction intervals. * *p* < 0.05, ** *p* < 0.01.

A total of 11 coronal sections, spaced 480 μm apart, were analyzed in each macaque brain (see [17]; Figure 3B). Results are reported in Figure 6A. When compared with the other 8 species previously studied with the same approach (Figure 6B), all primates displayed a significantly greater density of amygdala INs, with a one order of magnitude difference in comparison to rodents. The macaques had a cell density slightly lower than marmosets and chimpanzees (tabulated data can be found in Dataset S1). Similar to the cortex, the percentage of type 2 cells in the amygdala was very low, with the vast majority being represented by type 1 cells (pie chart in Figure 6A). The measure of average cell soma diameters was 4–8 µm for type 1 cells and 8–17 µm for type 2 cells. The estimated total number of scINs in the whole macaque amygdala was around 250,000.

**Figure 7:**
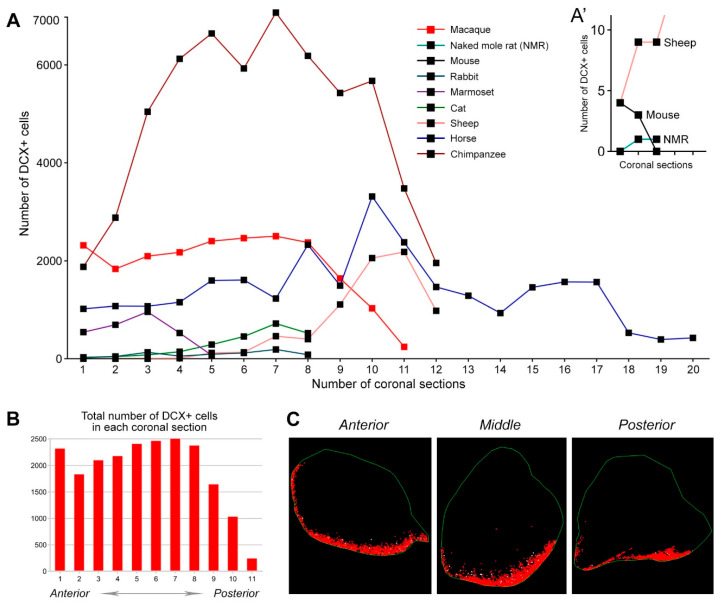
Spatial distribution of cINs in the macaque amygdala. (**A**) Anterior–posterior distribution of DCX^+^ cells in the entire length of the amygdala. The line plot indicates the mean total number of DCX^+^ cells counted in each coronal brain section of macaques (red; histogram in (**B**)), in comparison with other mammals (black; the number of coronal sections considered varies depending on the amygdala length and brain size; raw data in [17]). (**A’**) Enlargement of the line plot for rodents shows that they have just a few DCX^+^ cells in the amygdala. (**C**) Topographical distribution of DCX^+^ cells within the macaque amygdala obtained from Neurolucida by placing markers on cells in brain coronal sections used for cell counting (red dots: DCX^+^ cells; green line: amygdala perimeter). Most immature neurons are concentrated within the basolateral amygdala, particularly in the paralaminar nucleus.

As for the topographical distribution of the scINs in the amygdala, the anterior-posterior arrangement is indicated in the line plot of Figure 7A and in the histogram of Figure 7B. The arrangement in the coronal plane is shown in Figure 7C, with most DCX^+^ cells grouped within the basolateral amygdala with a marked concentration in the paralaminar nucleus.

Linear densities obtained from systematic quantification of the DCX^+^ cells were used to perform phylogenetic and linear regression analyses (Figure 6C,D). Ancestral character state reconstructions of trait evolution for DCX^+^ cell density mapped onto the phylogeny were performed in the amygdala (Figure 6C). Among the examined brains, the Euarchontoglires clade showed a strong distinction between its two orders, Primates (marmoset, macaque, chimpanzee) and Rodents, with the former exhibiting the highest cIN density and the latter the lowest cIN density. The Laurasiatheria clade (Carnivora, Artiodactyla, and Perissodactyla), meanwhile, occupies an intermediate position.

To explore the scaling relationship between DCX^+^ cell densities and brain size (scIN densities against brain weight; Figure 6D), least squares regression analysis was conducted using the data obtained here for macaques compared with a dataset previously reported for other young adult mammals [17]). The results indicated a significant positive association for the amygdala (R^2^ = 0.51, *p* = 0.030; Figure 6D).

## 4. Discussion

Recent research in comparative neuroplasticity has brought attention to a population of undifferentiated cells in arrested maturation—known as immature neurons (INs)—that are particularly abundant in the cerebral cortex and amygdala of large-brained, gyrencephalic species, thus suggesting that an evolutionary trade-off has occurred between different types of plasticity across widely different mammals [18,19,20]. To better define the phylogenetic variation of this novel cell population, our research extends the number of mammal species that are systematically quantified for INs, with a particular focus on primates. Furthermore, another urgent need is to characterize non-rodent animal models to allow in-depth and functional investigation of these cells, including in the neocortex, given that INs are restricted to the piriform cortex in laboratory rodents. Macaques, as relatively large-brained, gyrencephalic primates, represent an excellent model that is commonly used in biomedical research [41,42]. Here, we measured IN densities in the piriform cortex, neocortex, and amygdala of young adult rhesus macaques using a previously established method for comparing quantitative results among diverse mammals [15,16,17,26]. Our findings in macaques confirmed a series of morphological, cellular, and topographical features that have been consistently found to be shared by cortical and amygdala INs in a dozen mammal species, including mice and primates (including humans) [5,8,13,14,15,16,17,24,25,40,43,44,45,46]. We discuss our results in a comparative and evolutionary perspective.

### 4.1. Macaque Cortical and Subcortical INs in Mammal Phylogenetic Variation

The class of mammals, including nearly 6000 species, displays remarkable diversity in size, morphology, habitat, and behavior, with their brains reflecting this variability: from 0.6 g in mice to 5–10 kg in some large baleen whales [47,48,49]. Recent research in comparative neuroplasticity has revealed that widely different mammals are endowed with distinctive potential in structural plasticity, in terms of their types, amount, and anatomical location [20,50,51,52]. Particularly for neurogenic processes, it has been hypothesized that a trade-off has occurred during evolution between stem cell-driven adult neurogenesis, which is more active and extended in time in small-brained, lissencephalic rodents, and non-dividing, late-maturing INs, which are far more abundant in large-brained, gyrencephalic species, including primates [19,53]. Hence, INs appear to be an important form of structural plasticity, markedly varying in mammal brain evolution and increasing in the primate lineage that includes humans [20], thus raising interest in a translational perspective. However, the above-mentioned difference in cIN densities across mammals is more evident when considering the “extremes” represented by small-brained nocturnal (or subterranean) rodents, relying strongly on olfaction, compared to large-brained diurnal and social primates (see Section 4.2). Within this sample, statistically significant associations between cIN density and measures of brain size indicate that allometric scaling is an important contributor to interspecific variation. However, brain size alone does not fully explain the observed pattern. Notably, species such as cats, dogs, and rabbits exhibit relatively high cIN densities for their brain size, suggesting a phylogenetic shift within their lineages that is superimposed on broader scaling relationships. Thus, the current data are most consistent with a combination of allometric and phylogenetic influences on cIN density. Carnivora, Artiodactyla, and Perissodactyla belong to the clade Laurasiatheria, while Rodents and Primates are in the clade Euarchontoglires, their divergence occurring approximately 90 million years ago (Figure 5C; see Ref. [52]). The presence of high cIN density in both Laurasiatheria and Primates may reflect either independent evolutionary increases or, alternatively, a more ancient origin with subsequent reduction or loss in the rodent lineage. Resolving the relative contributions of factors such as brain size scaling, phylogenetic history, and ecological adaptations will require a broader comparative framework encompassing additional species and lineages, which remains an important objective of our ongoing research program.

**Figure 8:**
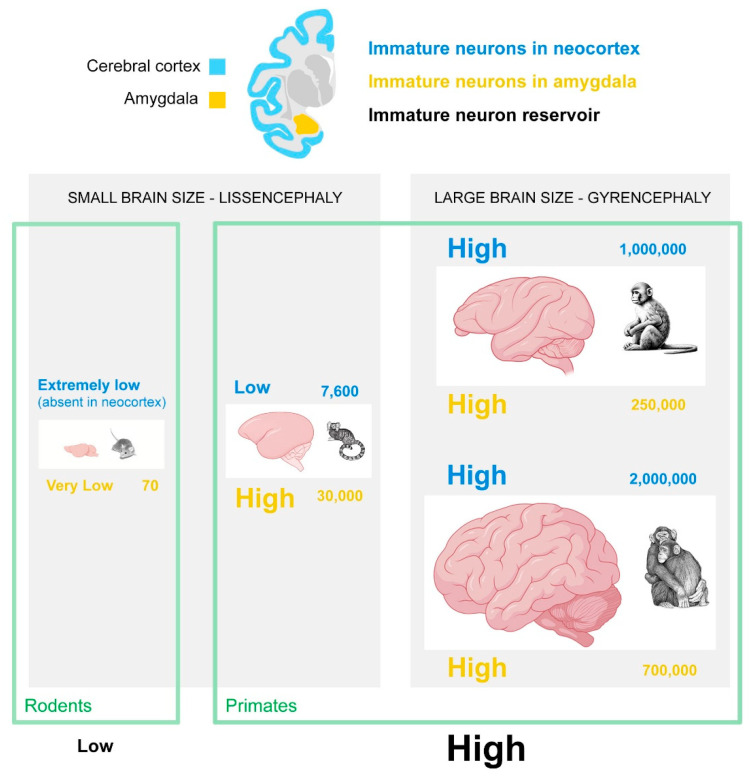
Schematic summary illustrating the amount of cortical immature neurons (blue) and amygdala immature neurons (yellow) in three primate species and the difference compared with rodents. “Low” and “high” refer to DCX^+^ cell densities, while numbers indicate the estimated total DCX^+^ cells/hemisphere. Small-brained, lissencephalic species have lower numbers of cINs. By contrast, all primates have large numbers of INs in the amygdala. Among primates, macaques are closer to chimpanzees than marmosets. Animal icons were reproduced with permission from [15]; this article is distributed under the terms of the Creative Commons Attribution License, which permits unrestricted use and redistribution provided that the original author and source are credited. Brain icons were created with BioRender.

Among primates, previous reports showed that the small-brained, lissencephalic marmosets have low numbers of cINs, while these cells are extremely abundant in the large-brained, gyrencephalic chimpanzees [15]. Here, we found that macaques, placed in the middle between marmosets and chimpanzees based on gyrification index and brain size (but closer to the latter; Figure 1C), have high numbers of cINs, very close to chimpanzees (Figure 5B), thus confirming a relationship between the size of this cell population and the expansion of neocortical structures through phylogeny [15,19]. Concerning the amygdala, previous reports described a prevalence of scINs in the primates examined to date (marmoset and chimpanzee) and here we show the same trend for macaques (Figure 6B). In brief, small-brained, lissencephalic species characterized by relatively small cortical mantles (including marmoset) have lower numbers of cINs, while all primates have large numbers of INs in the amygdala (summarized in Figure 8).

### 4.2. Evolutionary and Translational Considerations

Taking into account the nuanced, interrelated contributions of phylogenetic history, brain scaling, and ecological niche-related functions (see the section above), a pattern is emerging indicating a trade-off between stem cell-driven neurogenesis and non-dividing immature neurons across diverse mammals [18,19,20]. It is worth noting that the two types of structural (neurogenic) plasticity occur in different brain regions: the olfactory bulb and hippocampus for stem cell-driven plasticity and the cerebral cortex and amygdala for immature neurons [19,20,52]. This regional specialization has been suggested to be linked to neural functions induced by selective pressures of different ecological niches: nocturnal (subterranean) rodents have very large olfactory bulbs and rely more on olfaction and navigation for their survival, while diurnal (social) primates are characterized by expansion of the neocortex and basolateral amygdala and rely more on high-order cognitive functions and emotional regulation to mediate social interactions [19,54,55,56,57] (see below).

The findings of the present study also have important translational value, showing that macaques fit with the phylogenetic variation of both cINs and scINs. Notwithstanding ethical issues, primate models are essential for advancing fundamental knowledge in biomedical research [29]. Nonhuman primates have frequently been used in medical and scientific research due to their similarity with humans regarding physiology, neuroanatomy, reproduction, development, cognition, social complexity, genetics, aging, and disease [29,30,31,58,59,60]. The distribution and features of IN cell populations in macaques described here share key similarities with the currently available data in humans. First, it is confirmed that the cINs occur within layer II of the entire cortical mantle (although with some regional differences). Second, the topographical location of the INs in the basolateral amygdala appeared more concentrated in the paralaminar nucleus [17], thus very similar to that described in humans [14]. Overall, data from the present study indicates macaques as an ideal primate model for studying INs in view of human translation [41,42].

### 4.3. A Reservoir of Immature Neurons in High-Order Brain Regions of Primates

The present study on macaque brains confirms that primates endowed with relatively large brain size and gyrencephaly possess remarkable reservoirs of INs both in the amygdala and in cerebral cortex layer II, the latter extended to the entire neocortical mantle. In comparison, rodents studied to date have only a few immature cells in the amygdala, and the cINs are restricted to the piriform cortex. This research being still relatively novel in the neurosciences, in the absence of further knowledge on the physiological/functional role of INs, it is difficult to derive functional interpretations with certainty. Nevertheless, we suggest the following four broad conclusions: (i) the INs, as populations of non-dividing neuronal precursors in arrested maturation, have undergone an evolutionary trade-off with stem cell-driven neurogenesis (active in the neurogenic niches of the lateral ventricle and hippocampus) [19]; (ii) such recruitment has occurred in high-order brain regions, such as the cerebral cortex and the amygdala, thus going beyond the olfactory bulb and hippocampus and related functions [19,54]; (iii) in the amygdala the INs are located in the basolateral complex, namely its subdivision with strong cortical connections that has increased in size in primates [61,62,63]; (iv) in the cerebral cortex, the INs are present in the entire perimeter of layer II, namely a cortico-cortical association layer that has increased in thickness in certain species [64], likely representing a cross-cutting feature not linked to specific functions or areas. Thus, it is reasonable to hypothesize that a reservoir of undifferentiated neurons is maintained in high-order, highly interconnected regions of primate brains that play a crucial role in the sophisticated cognitive functions and complex sociality of these mammal species [55,56,57,65,66,67,68].

Although the field is still in its early stages, the study of INs in primate brains might open a wide range of possibilities, including an understanding of their roles in postnatal brain development and sculpting, in neurodevelopmental or psychiatric disorders, in aging, and potentially as a source of young, undifferentiated cells in disease and neurodegeneration.

## Figures and Tables

**Table 1:** Brain samples of male young adult rhesus macaques used in this study.

Species	Source	Specimens	Age (Years)	Fixation	Fixative	PMI
*Macaca mulatta*	JBSA Fort Sam Houston	4	7.4	Immersion	10% buffered formalin	<1 h
	JBSA Fort Sam Houston		7.6			
	Emory National Primate Research Center		8.0			
	Oregon National Primate Center		10.2			

## Data Availability

The raw data supporting the conclusions of this article will be made available by the authors, without undue reservation.

## Supplementary Materials

The following supporting information can be downloaded at https://www.mdpi.com/article/10.3390/cells15131158/s1. Figure S1: Positioning of macaque in the phylogenetic variation of cIN density in the neocortex of mammals. Figure S2: Ancestral character state reconstructions of trait evolution for DCX^+^ cell density mapped onto the phylogeny in cerebral cortex. Figure S3: Least squares regression of DCX^+^ cell density in whole cerebral cortex against brain size (brain weight). Table S1: Cell soma diameter range of type 1 and type 2 cells in the cerebral cortex and amygdala of different mammals, including previously published data and results of the present study on macaques. Dataset S1: Tabulated data (cell counting).

## References

[1] Gómez-Climent M.A., Castillo-Gómez E., Varea E., Guirado R., Blasco-Ibáñez J.M., Crespo C., Martínez-Guijarro F.J., Nácher J. (2008). A Population of Prenatally Generated Cells in the Rat Paleocortex Maintains an Immature Neuronal Phenotype into Adulthood. Cereb. Cortex.

[2] Bonfanti L., Nacher J. (2012). New Scenarios for Neuronal Structural Plasticity in Non-neurogenic Brain Parenchyma: The Case of Cortical Layer II Immature Neurons. Prog. Neurobiol..

[3] König R., Benedetti B., Rotheneichner P., O’Sullivan A., Kreutzer C., Belles M., Nacher J., Weiger T.M., Aigner L., Couillard-Després S. (2016). Distribution and Fate of DCX/PSA-NCAM Expressing Cells in the Adult Mammalian Cortex: A Local Reservoir for Adult Cortical Neuroplasticity?. Front. Biol..

[4] Rotheneichner P., Belles M., Benedetti B., König R., Dannehl D., Kreutzer C., Zaunmair P., Engelhardt M., Aigner L., Nacher J. (2018). Cellular Plasticity in the Adult Murine Piriform Cortex: Continuous Maturation of Dormant Precursors into Excitatory Neurons. Cereb. Cortex.

[5] La Rosa C., Parolisi R., Bonfanti L. (2020). Brain Structural Plasticity: From Adult Neurogenesis to Immature Neurons. Front. Neurosci..

[6] Benedetti B., Dannehl D., König R., Coviello S., Kreutzer C., Zaunmair P., Jakubecova D., Weiger T.M., Aigner L., Nacher J. (2020). Functional Integration of Neuronal Precursors in the Adult Murine Piriform Cortex. Cereb. Cortex.

[7] Benedetti B., Reisinger M., Hochwartner M., Gabriele G., Jakubecova D., Benedetti A., Bonfanti L., Couillard-Despres S. (2023). The Awakening of Dormant Neuronal Precursors in the Adult and Aged Brain. Aging Cell.

[8] Benedetti B., Couillard-Després S. (2022). Why Would the Brain Need Dormant Neuronal Precursors?. Front. Neurosci..

[9] Bonfanti L., Seki T. (2021). The PSA-NCAM-positive “Immature” Neurons: An Old Discovery Providing New Vistas on Brain Structural Plasticity. Cells.

[10] Gleeson J.G., Lin P.T., Flanagan L.A., Walsh C.A. (1999). Doublecortin is a Microtubule-Associated Protein and is Expressed Widely by Migrating Neurons. Neuron.

[11] Fudge J., Decampo D., Becoat K. (2012). Revisiting the Hippocampal-Amygdala Pathway in Primates: Association with Immature-appearing Neurons. Neuroscience.

[12] de Campo D.M., Cameron J.L., Miano J.M., Lewis D.A., Mirnics K., Fudge J.L. (2017). Maternal Deprivation Alters Expression of Neural Maturation Gene tbr1 in the Amygdala Paralaminar Nucleus in Infant Female Macaques. Dev. Psychobiol..

[13] Piumatti M., Palazzo O., La Rosa C., Crociara P., Parolisi R., Luzzati F., Lévy F., Bonfanti L. (2018). Non-newly Generated, “Immature” Neurons in the Sheep Brain Are Not Restricted to Cerebral Cortex. J. Neurosci..

[14] Sorrells S.F., Paredes M.F., Velmeshev D., Herranz-Pérez V., Sandoval K., Mayer S., Chang E.F., Insausti R., Kriegstein A.R., Rubenstein J.L. (2019). Immature Excitatory Neurons Develop During Adolescence in the Human Amygdala. Nat. Commun..

[15] La Rosa C., Cavallo F., Pecora A., Chincarini M., Ala U., Faulkes C.G., Nacher J., Cozzi B., Sherwood C.C., Amrein I. (2020). Phylogenetic Variation in Cortical Layer II Immature Neuron Reservoir of Mammals. eLife.

[16] Pattaro A., Ghibaudi M., Corrente C., Telitsyn N., Graic J.-M., Aresu L., Sherwood C.C., Bonfanti L. (2025). Phylogenetic Variation of Layer II Cortical Immature Neurons in Dog and Horse Confirms Covariance with Brain Size and Neocortical Surface. Brain Struct. Funct..

[17] Ghibaudi M., La Rosa C., Telitsyn N., Graïc J.-M., Faulkes C.G., Sherwood C.C., Bonfanti L. (2025). Multispecies Characterization of Immature Neurons in the Mammalian Amygdala Reveals their Expansion in Primates. PLoS Biol..

[18] Palazzo O., La Rosa C., Piumatti M., Bonfanti L. (2018). Do Large Brains of Long-living Mammals Prefer Non-newly Generated, Immature Neurons?. Neural Regen. Res..

[19] Bonfanti L., La Rosa C., Ghibaudi M., Sherwood C.C. (2024). Adult Neurogenesis and “Immature” Neurons in Mammals: An Evolutionary Trade-off in Plasticity?. Brain Struct. Funct..

[20] Ghibaudi M., Zanone A., Bonfanti L. (2026). Brain Structural Plasticity in Large-brained Mammals: Not Only Narrowing Roads. Neural Regen. Res..

[21] La Rosa C., Ghibaudi M., Bonfanti L. (2019). Newly Generated and Non-Newly Generated “Immature” Neurons in the Mammalian Brain: A Possible Reservoir of Young Cells to Prevent Brain Aging and Disease?. J. Clin. Med..

[22] Cushman J.D., Drew M.R., Krasne F.B. (2021). The Environmental Sculpting Hypothesis of Juvenile and Adult Hippocampal Neurogenesis. Prog. Neurobiol..

[23] Page C.E., Biagiotti S.W., Alderman P.J., Sorrells S.F. (2022). Immature Excitatory Neurons in the Amygdala Come of Age During Puberty. Dev. Cogn. Neurosci..

[24] Li Y.-N., Hu D.-D., Cai X.-L., Wang Y., Yang C., Jiang J., Zhang Q.-L., Tu T., Wang X.-S., Wang H. (2023). Doublecortin Expressing Neurons in Human Cerebral Cortex Layer II and Amygdala from Infancy to 100 Year-old. Mol. Neurobiol..

[25] Coviello S., Gramuntell Y., Klimczak P., Varea E., Blasco-Ibañez J.M., Crespo C., Gutierrez A., Nacher J. (2022). Phenotype and Distribution of Immature Neurons in the Human Cerebral Cortex Layer II. Front. Neuroanat..

[26] Pattaro A., Zanone A., Sherwood C.C., Bonfanti L., Ghibaudi M. (2026). Comparative Approach for Quantitative Cell Counting Studies in Widely Different Mammalian Brains. J. Vis. Exp..

[27] Wilson D.E., Reeder D.M. (2005). Mammal Species of the World: A Taxonomic and Geographic Reference.

[28] Capitanio J.P., Emborg M.E. (2008). Contributions of Non-human Primates to Neuroscience Research. Lancet.

[29] Phillips K.A., Bales K.L., Capitanio J.P., Conley A., Czoty P.W., ‘t Hart B.A., Hopkins W.D., Hu S.L., Miller L.A., Nader M.A. (2014). Why Primate Models Matter. Am. J. Primatol..

[30] Simmons H.A. (2016). Age-associated Pathology in Rhesus Macaques (*Macaca mulatta*). Vet. Pathol..

[31] Harding J.D. (2017). Nonhuman Primates and Translational Research: Progress, Opportunities, and Challenges. ILAR J..

[32] Saleem K.S., Logothetis N.K. (2012). A Combined MRI and Histology Atlas of the Rhesus Monkey Brain in Stereotaxic Coordinates.

[33] Paxinos G., Petrides M., Huang X., Toga A.W. (2008). The Rhesus Monkey Brain in Stereotaxic Coordinates.

[34] Mikula S., Trotts I., Stone J.M., Jones E.G. (2007). Internet-enabled high-resolution brain mapping and virtual microscopy. Neuroimage.

[35] Bakker R., Tiesinga P., Kötter R. (2015). The Scalable Brain Atlas: Instant web-based access to public brain atlases and related content. Neuroinformatics.

[36] Dubach M.F., Bowden D.M. (2009). BrainInfo Online 3D Macaque Brain Atlas: A Database in the Shape of a Brain. Society for Neuroscience Annual Meeting.

[37] Rohlfing T., Kroenke C.D., Sullivan E.V., Dubach M.F., Bowden D.M., Grant K.A., Pfefferbaum A. (2012). The INIA19 Template and NeuroMaps Atlas for Primate Brain Image Parcellation and Spatial Normalization. Front. Neuroinform..

[38] Kumar S., Stecher G., Suleski M., Hedges S.B. (2017). TimeTree: A Resource for Timelines, Timetrees, and Divergence Times. Mol. Biol. Evol..

[39] Luzzati F., Bonfanti L., Fasolo A., Peretto P. (2009). DCX and PSA-NCAM Expression Identifies a Population of Neurons Preferentially Distributed in Associative Areas of Different Pallial Derivatives and Vertebrate Species. Cereb. Cortex.

[40] Alderman P.J., Saxon D., Torrijos-Saiz L.I., Sharief M., Page C.E., Baroudi J.K., Biagiotti S.W., Butyrkin V.A., Melamed A., Kuo C.T. (2024). Delayed Maturation and Migration of Excitatory Neurons in the Juvenile Mouse Paralaminar Amygdala. Neuron.

[41] Prescott M.J. (2010). Ethics of Primate Use. Adv. Sci. Res..

[42] Aguilera B., Perez Gomez J., DeGrazia D. (2021). Should Biomedical Research with Great Apes be Restricted? A Systematic Review of Reasons. BMC Med. Ethics.

[43] Ghibaudi M., Marchetti N., Vergnano E., La Rosa C., Benedetti B., Couillard-Despres S., Farioli-Vecchioli S., Bonfanti L. (2023). Age-related Changes in Layer II Immature Neurons of the Murine Piriform Cortex. Front. Cell. Neurosci..

[44] Freixes J., Abdel-Rahman F.E.S., Nebbia R., Medina L., Desfilis E. (2025). Postnatal Plasticity in the Olfactory System of the Juvenile Swine Brain. Brain Struct. Funct..

[45] Freixes J., Desfilis E., Medina L. (2026). Postnatal Plasticity in the Paralaminar Nucleus of the Pallial Amygdala in Juvenile Swine Brain. Brain Struct. Funct..

[46] Torrijos-Saiz L.I., Ghibaudi M., Sharief M., Ljungqvist Brinson L., Alvarez-Buylla A., Garcìa-Verdugo J.M., Herranz-Pérez V., Sorrells S.F. (2026). Immature Excitatory Neurons in the Postnatal Ferret Paralaminar Nuclei and Their Relationship to the Amygdala Across Species. J. Comp. Neurol..

[47] DeFelipe J. (2011). The Evolution of the Brain, the Human Nature of Cortical Circuits, and Intellectual Creativity. Front. Neuroanat..

[48] Zilles K., Palomero-Gallagher N., Amunts K. (2013). Development of Cortical Folding During Evolution and Ontogeny. Trends Neurosci..

[49] Brecht M. (2025). Large Brains: Big Unknowns in Cellular Neuroscience. Curr. Opin. Neurobiol..

[50] Paredes M.F., Sorrells S.F., Garcia-Verdugo J.M., Alvarez-Buylla A. (2016). Brain Size and Limits to Adult Neurogenesis. J. Comp. Neurol..

[51] Parolisi R., Cozzi B., Bonfanti L. (2018). Humans and Dolphins: Decline and Fall of Adult Neurogenesis. Front. Neurosci..

[52] Pattaro A., Ghibaudi M., Zanone A., Cerrato V., Sherwood C.C., Bonfanti L. (2026). Mixed Signals and Interspecies Variation in the Plasticity of Adult Mammal Brains. Cells.

[53] Morizet D., Bally-Cuif L. (2025). Reduced Adult Neurogenesis in Humans Results From a Tradeoff Rather Than Direct Negative Selection. BioEssays.

[54] Aboitiz F., Montiel J.F. (2015). Olfaction, Navigation, and the Origin of Isocortex. Front. Neurosci..

[55] Roberts A.I., Roberts S.G.B. (2020). Communicative Roots of Complex Sociality and Cognition. Biol. Rev..

[56] Melchionna M., Castiglione S., Girardi G., Profico A., Mondanaro A., Sansalone G., Chatar N., Ramos A.P., Fernández-Monescillo M., Serio C. (2025). Cortical Areas Associated to Higher Cognition Drove Primate Brain Evolution. Commun. Biol..

[57] Magrou L., Joyce M.K.P., Froudist-Walsh S., Datta D., Wang X.-J., Martinez-Trujillo J., Arnsten A.F.T. (2024). The Meso-connectomes of Mouse, Marmoset, and Macaque: Network Organization and the Emergence of Higher Cognition. Cereb. Cortex.

[58] Stonebarger G.A., Bimonte-Nelson H.A., Urbanski H.F. (2021). The Rhesus Macaque as a Translational Model for Neurodegeneration and Alzheimer’s Disease. Front. Aging Neurosci..

[59] Shively C.A., Clarkson T.B. (2009). The Unique Value of Primate Models in Translational Research. Am. J. Primatol..

[60] Chiou K.L., Montague M.J., Goldman E.A., Watowich M.M., Sams S.N., Song J., Horvath J.E., Sterner K.N., Ruiz-Lambides A.V., Martínez M.I. (2020). Rhesus Macaques as a Tractable Physiological Model of Human Ageing. Philos. Trans. R. Soc. Lond. B Biol. Sci..

[61] Barton R.A., Aggleton J.P., Grenyer R. (2003). Evolutionary Coherence of the Mammalian Amygdala. Proc. Biol. Sci..

[62] Chareyron L.J., Banta Lavenex P., Amaral D.G., Lavenex P. (2011). Stereological Analysis of the Rat and Monkey Amygdala. J. Comp. Neurol..

[63] Chin R., Chang S.W.C., Holmes A.J. (2023). Beyond Cortex: The Evolution of the Human Brain. Psychol. Rev..

[64] de Sousa A.A., Rigby Dames B.A., Graff E.C., Mohamedelhassan R., Vassilopoulos T., Charvet C.J. (2023). Going Beyond Established Model Systems of Alzheimer’s Disease: Companion Animals Provide Novel Insights into the Neurobiology of Aging. Commun. Biol..

[65] Krubitzer L. (2009). In Search of a Unifying Theory of Complex Brain Evolution. Ann. N. Y. Acad. Sci..

[66] Sherwood C.C., Gómez-Robles A. (2017). Brain Plasticity and Human Evolution. Annu. Rev. Anthropol..

[67] Putnam P.T., Chang S.W.C. (2021). Social Processing by the Primate Medial Frontal Cortex. Int. Rev. Neurobiol..

[68] Preuss T.M., Wise S.P. (2022). Evolution of Prefrontal Cortex. Neuropsychopharmacology.

