# Supplementary material for: Quantitative and Phylogenetic Analyses of Immature Neurons in Cortical Layer II and Amygdala of Macaque Monkeys
Source: Cells. 2026 Jun 25;15(13):1158. doi: 10.3390/cells15131158 (PMC13359871; doi:10.3390/cells15131158)
Supplement: Supplementary file 1 [file cells-15-01158-s001.zip › cells-4347818-supplementary.pdf]

Quantitative and phylogenetic analyses of immature neurons in cortical layer II and amygdala of macaque monkeys

Alessia Pattaro, Marco Ghibaudi, Madeline Bramel, Chet C. Sherwood, Luca Bonfanti

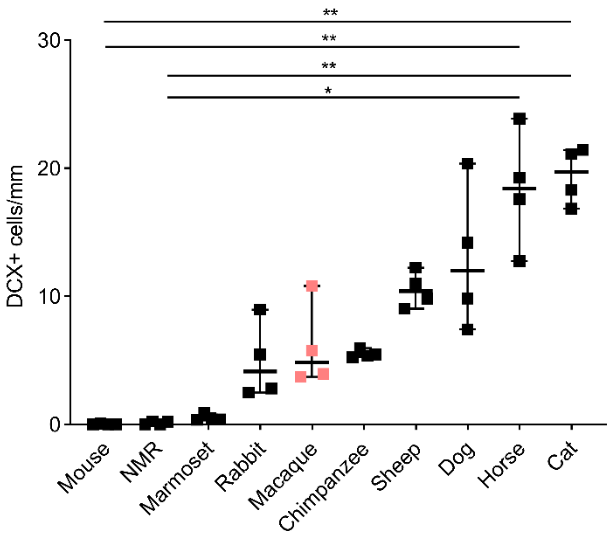

Figure S1 - Positioning of macaque in the phylogenetic variation of cIN density in the neocortex of mammals; data obtained in the present study (in pink) are compared with data previously reported [15,16]; in black). Macaques are placed among the higher density scores, and a significant difference is maintained between rodents and gyrencephalic species. NMR, naked mole rat.

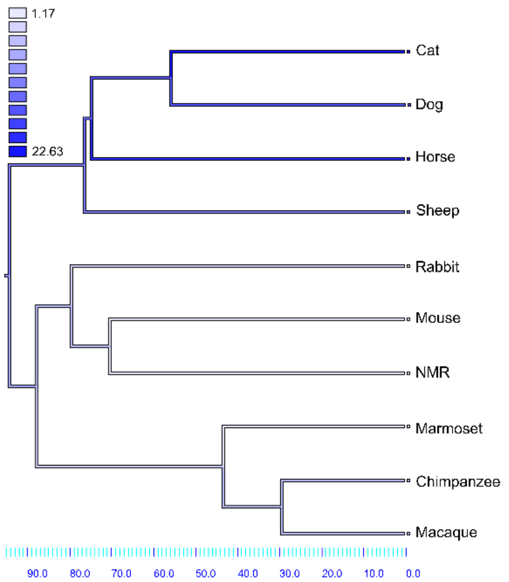

Figure S2 – Ancestral character state reconstructions of trait evolution for DCX$^+$ cell density mapped onto the phylogeny, in cerebral cortex.

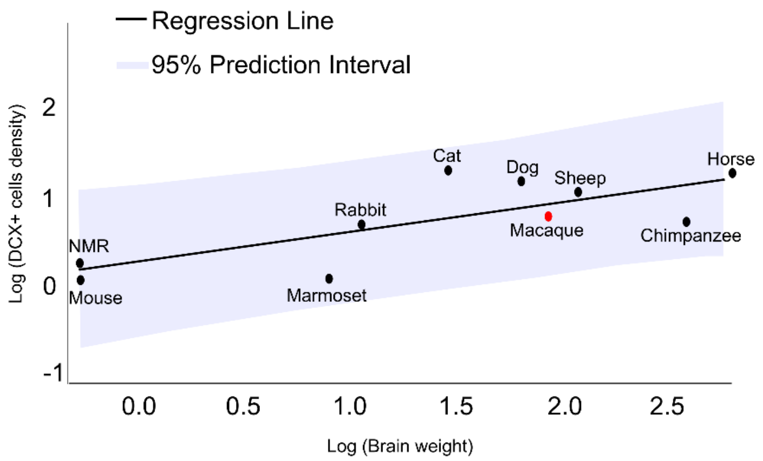

Figure S3 - Least squares regression of DCX^+ cell density in whole cerebral cortex against brain size (brain weight). The regression plot is on a log scale and shows the 95% prediction intervals.

Table S1 – Range of cell soma diameters of type 1 and type 2 DCX^+ cells in the cerebral cortex and amygdala of different mammals, including previously published data and results of the present study on macaques. NMR, naked mole rat.

**Species**	**IN cell soma diameter in cortical layer II** (µm) (La Rosa et al., 2020b and Pattaro et al., 2025)		**IN cell soma diameter in amygdala** (µm) (Ghibaudi et al., 2025b)	
	**Type 1**	**Type 2**	**Type 1**	**Type 2**
Mouse	4-8	8-13	3-8	-
NMR	3-8	8-13	4-9	-
Marmoset	3-9	9-16	4-8	9-15
Rabbit	4-9	9-16	5-9	9-15
Sheep	3-7	7-13	5-9	9-16
Cat	4-7	7-16	4-9	9-14
Chimpanzee	3-9	9-17	6-9	9-19
Dog	4-9	9-19	-	-
Horse	4-9	9-20	4-9	9-18
Macaque	3-8	8-13	4-8	8-17

Dataset S1 (Excel file with data from cell counting – provided separately)
